# Increasing Awareness and Use of Iodised Salt in a Marginalised Community Setting in North-West Pakistan

**DOI:** 10.3390/nu7115490

**Published:** 2015-11-23

**Authors:** Nicola Lowe, Elizabeth Westaway, Akhtar Munir, Saba Tahir, Fiona Dykes, Monique Lhussier, Mick McKeown, Michael Zimmerman, Maria Andersson, Sara Stinca, Mukhtiar Zaman

**Affiliations:** 1 International Institute of Nutritional Sciences and Applied Food Safety Studies, University of Central Lancashire, Preston PR1 2HE, UK; elizabeth.westaway@gmail.com; 2 Abaseen Foundation, Peshawar 25000, Pakistan; akhtar_psw@yahoo.com (A.M.); saba_ktk85@yahoo.com (S.T.); 3 Maternal and Infant Nutrition and Nurture Unit (MAINN), University of Central Lancashire, Preston PR1 2HE, UK; FCDykes@uclan.ac.uk; 4 Department of Public Health and Wellbeing, Faculty of Health and Life Sciences, Northumbria University, Newcastle Upon Tyne NE7 7XA, UK; monique.lhussier@northumbria.ac.uk; 5 School of Nursing, University of Central Lancashire, Preston PR1 2HE, UK; MMckeown@uclan.ac.uk; 6 Human Nutrition Laboratory, Institute of Food, Nutrition, and Health, ETH Zurich, 8092 Zurich, Switzerland; michael.zimmermann@hest.ethz.ch (M.Z.); maria.andersson@hest.ethz.ch (M.A.); sara.stinca@hest.ethz.ch (S.S.); 7 Khyber Teaching Hospital, Khyber Medical University, Peshawar, Pakistan; mza38@hotmail.com

**Keywords:** iodine deficiency, goitre, urinary iodine concentration, community engagement, iodised salt, Pakistan, Khyber Pakhtunkhwa

## Abstract

Iodine deficiency is still prevalent in parts of Pakistan, despite the introduction of a national Iodine Deficiency Disorder Control Programme in 1994. The purpose of this study was to gain an understanding of the knowledge, attitudes and practice regarding the use of iodised salt in a brick kiln community, and to use this information to design an intervention to increase its consumption. A cross-sectional survey was used to assess the use of iodised salt and focus group discussions explored the attitudes and barriers to its use. Thematically analysed transcripts informed the design of a 4-month intervention. Iodised salt sales and urine iodine concentration (UIC) were monitored to assess the effectiveness of the intervention. At baseline, 2.6% of households reported use of iodised salt and barriers included its higher cost and belief about a negative impact on reproduction. During the intervention, sales of salt labelled as iodised increased by 45%, however this was not reflected in an increase in UIC. This study highlighted the positive impact of education and awareness raising on iodised salt consumption in a hard to reach, marginalised community. However, issues regarding adequate iodisation by local producers and appropriate storage also need to be urgently addressed at a provincial level.

## 1. Introduction

Iodine deficiency is one of the most common, yet easily preventable causes of brain damage worldwide, and is of international public health concern, particularly in developing countries [1,2]. Iodine is required for thyroid hormone synthesis, therefore the consequences of inadequate iodine intake include a spectrum of adverse effects on physical and mental growth and development, collectively referred to as the Iodine Deficiency Disorders (IDD). The social and economic impact of iodine deficiency is significant, with iodine deficiency resulting in lower IQ, productivity and student achievement.

Monitoring iodine status at the population level is now frequently included in nutrition surveys. Current estimates (2011), based on urinary iodine concentration (UIC) of school-aged children as a biomarker of recent iodine intake [3] indicate that, overall, 29.8% of the world’s population has an inadequate iodine intake. Globally, inadequate iodine intake is greatest in parts of Europe, Africa and South East Asia.

The use of iodised salt in IDD prevention, particularly in iodine deficient regions, was first recommend by the World Health Organization (WHO) in 1952 [4] and is recognised as efficient and cost-effective. However, in order to be effective, it needs to reach vulnerable members of the population (*i.e.*, pregnant women and children) and the salt needs to be adequately iodised (15–40 ppm iodine content) [3]. The national survey in 2011 indicates that 69% of households in Pakistan use iodised salt, however there are still regions where adequately iodised salt is not available, and/or not accepted by the communities. The amount of iodine added to salt during the fortification process needs to account for the loss of iodine during transport and storage and should be regularly monitored by government agencies. In addition, the use of iodised salt in the food manufacturing industry is not widespread and has not been the focus of government initiatives [5].

Pakistan’s national Iodine Deficiency Disorders Control Programme (IDDCP) was launched in 1994 but despite this approximately half of Pakistan’s population of 200 million is affected with IDD [2]. The National Nutrition Survey in Pakistan revealed marked provincial variation; for example, within Khyber Pakhtunkhwa, a north western province characterised by pockets of high levels of deprivation and low average educational status, 25.7% of children aged 6–12 years are at risk of iodine deficiency [6].

The study presented in this paper was undertaken in a marginalised, rural, brick kiln community located on the outskirts of Peshawar in Khyber Pakhtunkhwa comprised of 5000 households in an area of approximately 5 square miles. This is close to the federally administered tribal areas (FATA) and the population is composed of Pakistani and Afghan refugees with a traditional tribal culture which includes the implementation of a Jirga system to maintain law and order in the community, alongside the mainstream government systems. The Jirga is an informal gathering of men that has a key governing and decision-making role in the Pushtun community. The Jirga takes on a number of roles that can include conflict resolution, information dissemination and gathering, alliance building, peace-keeping, problem-solving and community development. This research was undertaken as part of a broader study of the role of the Jirga in community engagement of health-related research and was conducted in collaboration with the Abaseen Foundation (AF), a local non-governmental organization (NGO) implementing health care and education projects. Within this project, a survey of 1043 homes was undertaken to assess the health care needs of the brick kiln community. It revealed that 97% of the households surveyed were unaware of the benefits of iodised salt.

The purpose of this study was to explore in more detail the knowledge, attitudes and practice relating to use of iodised salt in this community, and to use this information to design and conduct an intervention to increase its consumption.

## 2. Methods

**Figure 1:**
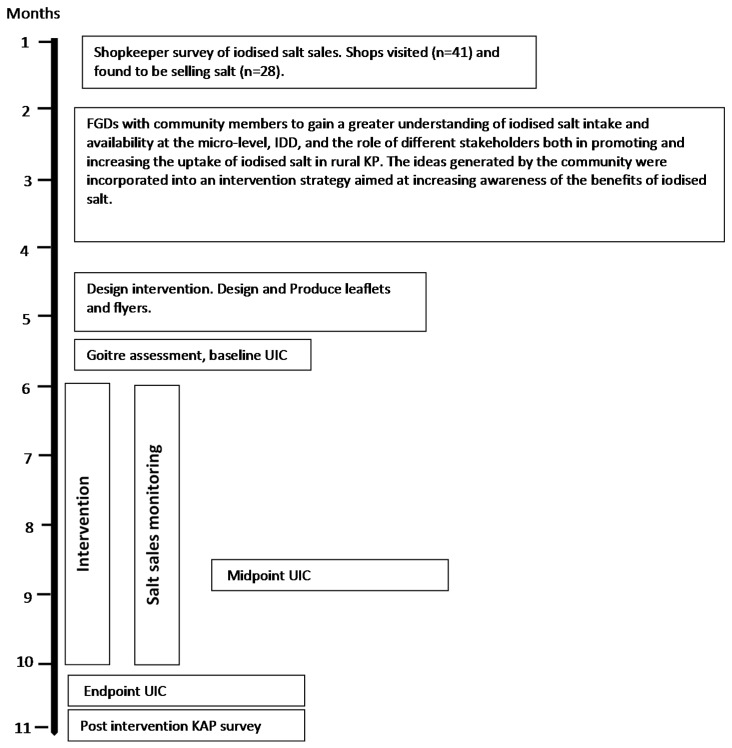
Overview of project timeline (FGD, Focus Group Discussion; UIC, Urine Iodine Concentration; IDD, Iodine Deficiency Disorders; KP, Khyber Pakhtunkhwa; KAP, Knowledge, Attitude and Practice).

Ethical approval for this research was granted by the respective ethics committee at the University of Central Lancashire, UK, and the Khyber Medical University, Peshawar, PK. An overview of the steps used in the design, implementation and monitoring of the intervention are illustrated in Figure 1.

### 2.1. Baseline Shopkeeper Survey and Sales Monitoring

At baseline, all 41 shops in the local bazaar were visited and 28 were found to sell iodised salt. These 28 shopkeepers were invited to participate in a survey on iodised salt. A semi-structured questionnaire was used to collect data on issues relating to personal and consumer knowledge and use of iodised salt. Shopkeepers were visited on a monthly basis for the duration of the intervention (described below) and data was collected on the quantities of different types of salt sold, as well as salt packaging and storage, including the display location.

### 2.2. Intervention Design and Participant Recruitment

The design of the intervention was developed following four focus group discussions (FGDs), conducted with Jirga Members and other community elders (*n* = 9), women over 40 years old (*n* = 8), women under 40 years old (*n* = 10) and male informal healthcare providers (*n* = 8). Participants were purposively selected based on their position within the community and their knowledge and experience of the community. Audiotaped FGDs were conducted in Pushtun, transcribed and translated into English and then thematically analysed using the the thematic networks analysis approach [7]. The details of this process are outside the scope of this paper and will be described elsewhere. The outcome of this analysis, together with information from the behaviour change literature [8], facilitated the design of a 4-month intervention. This intervention included the distribution of leaflets and posters designed by the research team, based on the United Nations International Children’s Emergency Fund, Information, Education and Communication material (UNICEF IEC) and written in Urdu, outlining the consequences of iodine deficiency and promoting the use of iodised salt. The UNICEF IEC material was adapted by replacing the pictures with more culturally appropriate pictures for this conservative community. These were distributed at the Mosque, Hujra (venue for social gathering of men), local health centres and schools, to shopkeepers and informal health care providers, and at households, brick kilns and communal areas. The leaflets and posters were monitored on a monthly basis and a total of 9563 leaflets and 496 posters were distributed over the intervention period. Since many of the adult community members are illiterate, verbal health education sessions were also given to groups (Jirga members, health care providers, school teachers and school children) by members of the research team. In addition, to reach females who do not move outside the family compound very often, door to door visits were made by trained social mobilisers to offer education sessions either to individuals or to small groups within the home. These health education sessions included information regarding sources of iodine; iodine deficiency statistics; iodine requirements; iodine-related health problems; benefits of iodised salt; use of iodised salt.

In order to assess the population iodine status, a sample of 300 boys aged 6–12 years from four primary schools in the brick kiln community (situated within a 5 km radius of the Health Centre) were invited to provide a spot urine sample on three occasions at baseline, midpoint and end of the intervention period. In this conservative Muslim community, the recruitment of boys into a study such as this is much more culturally acceptable than girls therefore, only boys were invited to participate in the study. The boys were given a participant information sheet and consent form to take home and show or read or have read to their parents or guardian. The consent form was signed or marked with a cross to show that the parent or guardian understood and gave informed consent for their child to participate in the study and provide urine samples. The boys were told on which date the researchers would come to their school. During the morning, participants were asked to give a urine sample. Each urine sample was labelled with the participant’s identification number, as well as the date and time of collection. Samples were transferred to a cool box for transportation back to the Khyber Teaching Hospital laboratory in Peshawar where they were stored at −20 °C prior to transportation to the Eidgenössische Technische Hochschule (ETH) Zurich for analysis. UIC was measured using a modification of the Sandell-Kolthoff reaction with spectrophotometric detection [9]. The coefficient of variation for UIC (±SD) in this laboratory is 11.5% at 31 ± 4 µg/L and 3.6% at 212 ± 8 µg/L [10].

The boys were also examined at baseline for the presence of palpable goitre (grade 0, 1 or 2) or visible goitre by an experienced medical doctor. Each child was given a grade, where grade 0 indicates no visible or palpable goitre, grade 1 is palpable but not visible, and grade 2 is visible and palpable. Total Goitre Rate (TGR) was calculated for this sample, where TGR = (Grade 1 + Grade 2)/number examined [3].

### 2.3. Intervention Effectiveness

The effectiveness of the intervention was assessed using quantitative and qualitative indicators. Quantitative indicators were assessed by monthly monitoring of salt sales from the 28 shops in the local bazaar, and by measuring the UIC of boys attending the local schools at baseline, midpoint and endpoint of the intervention, as described above.

Qualitative indicators of knowledge, attitude and practice were assessed in a KAP survey which was conducted with a sample of 50 households from the original household survey (5%). Households were selected based on a cluster sampling technique. The KAP survey was composed of 68 semi-structured questions, exploring three key themes: (1) current knowledge about the consequences of iodine deficiency and the benefits of iodised salt, and if this had changed since the start of the intervention; (2) sources of information about the health benefits of iodised salt; (3) current use of iodised salt in the household, where it was purchased, and current attitudes amongst family members concerning its use. The questionnaire also included an observation checklist for the interviewer to complete (*i.e.*, the type and amount of salt visible in the household, and how it was packaged and stored). The survey was read out to respondents and the responses recorded by the interviewer.

### 2.4. Statistical Analyses

A Kolmogorov-Smirnov test revealed that the UIC data were not normally distributed, therefore a Kruscal-Wallis test was used to investigate potential differences in UIC at the three time points. Where significant differences were indicated, these were explored with a Mann-Whitney post hoc test with Bonferroni correction such that *p* ≤ 0.0167 was considered statistically significant.

## 3. Results

### 3.1. Baseline Shopkeeper Survey and Sales Monitoring

At baseline, the survey of shopkeepers revealed that they were all aware that iodised salt was commercially available. Three types of salt were routinely sold in their shops, Anwar Iodised salt, Shafaf salt (non-iodised) and Simple salt (non-iodised). The majority of the shopkeepers (67.9%) routinely stocked iodised salt, with 64.3% having it in stock on the day of the survey. When asked about the health benefits of iodised salt, 71.4% were aware that it helped to prevent goitre. No other health benefits were identified by the shopkeepers, and 28.6% were completely unaware of any health benefits. Sales and prices of the different types of salt are shown in Table 1. The retail price of the salt did not change during the study.

**Table 1:** Monthly salt sales and prices reported by shopkeepers during the baseline survey.

Type of Salt	Retail Price Range (Rp)	Number of 0.5 kg Packs Sold per Month
Anwar Iodised	20–22	1550
Shafaf	10–12	2913
Simple	6–10	4213

When asked their opinion of why iodised salt was not as popular as non-iodised salt, 35.7% (10/28) of the shopkeepers reported that it was believed to have a negative impact on reproduction, and two additional shopkeepers reported a general negative perception of the iodised salt. 37.5% (10/28) said that it is because it is too expensive and 21.4% (6/28) of shopkeepers did not know or did not give an answer.

**Figure 2:**
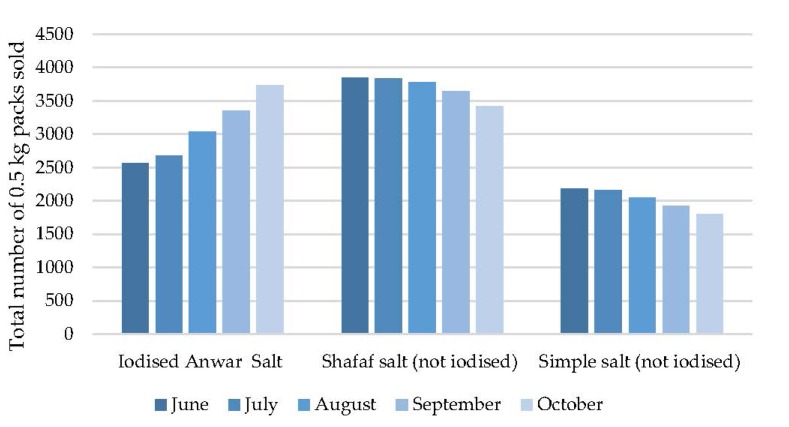
Salt sales during the intervention.

Monthly salt sales from the 28 shops selling iodised salt at the local bazaar from the start of the intervention (June 2013) to a month beyond the end of the intervention (October 2013) are shown in Figure 2.

The salt sales in the first month of the intervention already show some differences to the sales figures provided by the shopkeepers in the baseline survey, with simple salt sales in June 2013 already down by 50% of baseline values, and Anwar salt up by 66%, suggesting an early change in household purchasing behaviour. During the intervention period (June to October) the sales of Anwar Iodised salt increased by 45.1% in October compared with June, while the sales of Shafaf and simple salt decreased by 11.3% and 17.7% respectively. At all monthly time points between June and October, salt was found to be stored either inside or in front of the shop, in a dry place. The simple salt was sold in clear plastic bags with the salt visible, and the Shafaf and Anwar salt were sold in opaque, plastic bags (Figure S1 Supplementary Material).

### 3.2. Goitre Examination and UIC

The results of the examination for palpable goitre and UIC in 251 boys attending the four local primary schools are shown in Table 2. The TGR was 12.8% and visible goitre was 2.0%. Although 300 boys were invited to participate in the study, data and samples could only be collected from the boys that were present at school on the study day. School attendance is often inconsistent in this community due to frequent illness or prioritizing working on the brick kilns to contribute to the family income. This is reflected in the participant (*n*) numbers shown in Table 2.

The Median UIC at all three time points indicate mild iodine deficiency at a population level, using the cut-off value of 50–99 µg/L [3]. The Kruskal-Wallis test indicated that there were significant differences in the median UIC between the three time points. A Mann-Whitney post hoc test revealed that the median UIC at the endpoint was significantly lower than at the baseline and midpoint, *p* < 0.01. However, baseline and midpoint values were not significantly different. The UIC frequency distribution at each time point for the different categories defined by the WHO cut-off values from normal to severe deficiency are shown in Figure S2 (Supplementary Material) [3].

**Table 2:** Age, presence of goitre and urine iodine concentration (UIC) of the study participants.

	Mean	Median		Range (min-max)
Age (years) (*n* = 251)	7.6	7.0		6–12
Palpable Goitre	Number			%
Grade 0	219			87.3
Grade 1	27			10.8
Grade 2	5			2.0
Visible Goitre	5			2.0
UIC (µg/L)	Mean		Median	Range (min-max)
Baseline (*n* = 179)	81.75		75.18	13.83–291.34
Midpoint (*n* = 176)	93.39		77.86	5.86–320.33
Endpoint (*n* = 219)	71.06		59.80*	5.61–405.49

Note baseline: 2 samples removed due to high analysis coefficient of variation; Midpoint: 11 samples removed due to high analysis coefficient of variation; End: 3 samples removed due to high analysis coefficient of variation and 1 outlier removed. * Kruskal-Wallace with Mann-Whitney post hoc test indicates a significant difference compared with baseline and midpoint, where *p* < 0.016.

### 3.3. Knowledge, Attitude and Practice Survey

The characteristics of the respondents of the KAP survey are summarised in Table S1

(asupplementary material). The sample had an equal number of male and female respondents, none of whom were literate. The majority of the men that were interviewed were aged over 56, and the majority of the women were aged 46 to 55 years. An equal number of men and women reported listening to the radio, but only men watched television because it is only available at the Hujra. The households interviewed included both those with and without a son who had participated in the urine collection/goitre examination.

#### 3.3.1. Current Knowledge about the Consequences of Iodine Deficiency and the Benefits of Food Sources of Iodine and of Iodised Salt, and if this had Changed since the Start of the Intervention

The KAP survey showed that 100% of respondents reported that their knowledge had changed since the start of the intervention, with 26% (2 male and 10 female) reporting that they knew about the benefits and food sources of iodine, and the remaining 74% (22 male, 15 female) stating that they knew about iodine but not food sources. Seventy percent (17 male, 18 female) of the respondents knew that iodine deficiency resulted in goitre, and 30% (8 male, 7 female) knew that in addition to goitre, physical and mental development could be impaired.

None of the respondents knew of any individuals who had experienced symptoms of iodine deficiency since the start of the intervention. When asked if they felt at risk of developing iodine deficiency, 24% of respondents said yes, 28% said no, and 48% did not know. Twenty Eight percent of respondents felt that iodine deficiency was serious, 20% that it was not serious, and 26% did not know.

#### 3.3.2. Sources of Information about the Health Benefits of Iodised Salt

**Figure 3:**
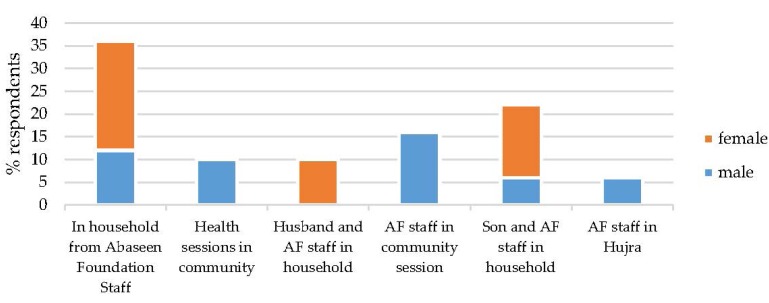
Sources of information regarding the health benefits of iodine.

The sources of information for females and males are shown in Figure 3. One hundred percent of respondents reported that iodized salt had been discussed in the Household since June 2013, with 70% reporting that they had also discussed iodine deficiency.

**Figure 4:**
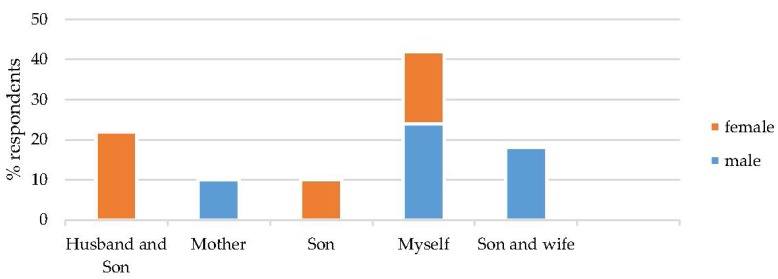
Which household members discussed iodized salt?

Figure 4 illustrates the responses from the male and female interviewees regarding who discussed iodized salt within the household. Women stated that they, their husbands and their sons were primarily involved in the discussions. The men stated that their mothers, sons, wives and themselves were primarily involved in the discussions.

#### 3.3.3. Current Use of Iodised Salt in the Household, where it was Purchased, and Current Attitudes amongst Family Members concerning its Use

Salt is used in bread (chapatti, roti) making and during cooking. It is stored in the kitchen, usually in a jar (glass, clay or tin) or in its original packaging. Men are responsible for buying salt for the household, with the women stating that either their son (52%) or their husband (48%) purchases the salt. Most of the households (96%) were currently using iodised salt, which was confirmed by the interviewer’s observation, and 90% acquired their salt from the local market. All of the respondents confirmed that they will use iodised salt in the future because they are aware of its health benefits and none reported a resistance from household members regarding its use.

The KAP survey revealed that female and male respondents most frequently named doctors as their most trusted source of information about the importance of using iodised salt (Figure S3 Supplementary Material). AF staff and school teachers were also well trusted by women, and religious leaders by men. Women believed that the best ways of providing health education about iodised salt was through sessions with men or household visits (Figure S4 Supplementary Material). Men also believed that household visits were effective, along with dissemination through religious leaders. Both men and women felt that the most important factors in encouraging community members to increase their use of iodised salt was through motivation (explaining the benefits) (32%) and awareness raising (38%). In terms of enabling the community to use iodised salt, ease of availability and lower price were reported as the most important factors (32% and 28% respectively).

## 4. Discussion

A comprehensive household survey was conducted by the AF prior to this study to determine the health care needs of this “Brick kiln” community. This survey revealed that, despite a national salt iodisation programme, this community was unaware of the benefits of iodised salt, and most of the households used non-iodised salt in their food preparation. The present study was attempting to increase health literacy levels with regards to the importance of iodized salt, in spite of low local education levels. Sørensen *et al.* [11] define health literacy as a concept which: “entails people’s knowledge, motivation and competences to access, understand, appraise, and apply health information in order to make judgements and take decisions in everyday life concerning healthcare, disease prevention and health promotion to maintain or improve quality of life during the life course.” In order to respond to health literacy needs, the WHO [12] calls for action at different levels: to ensure better health communication; to create and strengthen health literacy- friendly settings; and to develop policies for health literacy. These actions should be integrated to empower and enable people to make sound health decisions in the context of their everyday life [13]. The intervention described here, with a particular focus on community engagement, is situated within this global initiative to improve health literacy.

Following consultation with the Jirga, community engagement methods were used to design an intervention, aimed at increasing awareness of the health benefits of iodine in the diet, and increasing use of iodised salt at the domestic level. This broad approach was intended to result in interventions that were sensitive to local culture and consequently enhance impact. The intervention included group education sessions with different cadres of the community in schools, health centres and Hujras, door-to-door visits and poster and leaflet distribution. The KAP survey showed that the intervention was highly successful in increasing knowledge of the health benefits of iodised salt, with 100% of those interviewed stating that they were aware, compared with 3% at baseline. Female respondents most frequently cited sources of information as door-to-door sessions conducted by AF staff, coupled with information brought home by their husband and/or their sons, with male respondents most frequently citing community education sessions (Figure 3). In addition, female and male respondents stated that doctors and AF workers could be trusted and were reliable sources of information. Another study conducted in a remote rural part of Khyber Pakhtunkhwa by the Agha Khan Health Services also reports on their effectiveness in educating and motivating communities regarding the use and benefits of iodised salt. Education sessions were given at the village health centres, village social and religious gatherings, during immunization services and by motivating the people with the help of community health workers and traditional birth attendants. This area also had low literacy rates, and no direct television or radio transmission therefore these media channels were unable to play a role in promoting the use of iodized salt [14].

In the current study, interestingly, the posters and leaflet distributions were not mentioned at all as a source of information, which reflects the low literacy rates in the community. In fact, 100% of KAP survey respondents stated that they were not able to read (Table S1). Within the household, it appears that all female and male members were involved in the discussions about iodised salt (Figure 4). Food shopping is undertaken by male household members only, with women being restricted to the family compound, except for visits to the doctor or school, or on family occasions, when they must be accompanied by a male relative. The salt sales data collected from the shop keepers concurs with the survey data showing an increase in iodised salt sales of 45% after the intervention, compared with baseline. Similarly, the baseline survey indicated that 2.6% of households used iodised salt, whereas the post intervention KAP survey and visual inspection revealed that 96% were using iodised salt. While the results from the KAP survey could be partly explained by social desirability biases, this increase in sales suggests that the intervention has been effective. Whilst causality in its strict sense cannot be established, health literacy did seem to improve concerning the use of iodised salt, in spite of its price. The leverage of community resources, building on trust and mutual respect with key community members therefore proved successful.

In terms of optimising efforts for maintaining the message, for the women, household visits by social mobilisers, education of children in school, and informing the male household members in community sessions were fruitful. Men would also include informing the religious leaders who can reinforce the message through the Mosque.

The results of the baseline goitre assessment and urinary analysis indicate mild iodine deficiency in the Brick Kiln community. The National Nutrition Survey, Pakistan [6] reported that in Khyber Pakhtunkhwa province overall, children are iodine sufficient with a median UIC of 136.4 µg/L and goitre was found in only 0.2% children. In contrast, the iodine status of the boys in community was worse, with a median UIC at baseline of 75.18 µg/L and only 18.2% of boys had a UIC in the normal range (>100 µg/L), with 45.6% and 29.7% in the mild (50–99 µg/L) and moderately (20–49 µg/L) deficient categories respectively [15]. The UIC data suggesting low iodine intake and mild deficiency are supported by the elevated TGR of 12.8%. It is likely that prevalence of iodine deficiency in other members of the community (particularly women) is higher than that suggested by the data collected from boys. Data from the National Nutrition Survey, Pakistan [6] reveals that the number of women with UIC’s in the mild, moderate and severe deficiency categories is higher than that of the children nationally. Preliminary evaluation of the local diet (not reported here) revealed that the diet is low in natural sources of iodine, with fish rarely or never consumed, thus iodine deficiency is clearly a serious cause for concern in this community and is likely to have a long-term impact on the growth and cognitive development of the children living here. Despite the success of the intervention in increasing the knowledge and practice of using iodised salt, surprisingly, there was no improvement in the UIC values. It is possible that more time is needed in order for changes in intake to be reflected in the UIC, however there was no evidence of a trend towards an increase in UIC within the 4 month intervention period, in fact there appeared to be significant decrease. Other studies have also revealed a lack of association between self-reported iodized salt use and urinary iodine concentration [16], which may be due to consumption of meals outside of the home and processed foods that have not been prepared with iodised salt [5]. This is unlikely to be the case in the present study, since meals are rarely consumed outside of the home, particularly by children and all food is prepared from the basic ingredients within the household, including chapatti and roti. The WHO has recently published new guidelines on the fortification of salt, based on the salt consumption of the population and against the backdrop of the concurrent need to reduce overall salt consumption to less than 5 g per day [17]. For this community, the key issue is ensuring that the fortified salt is delivered safely within the acceptable dosage range. Ali *et al.* [18] reported similar UIC values to the present study in a rural region of North-West Pakistan, namely Gilgit and Hunza provinces, traditionally associated with a high prevalence of endemic goitre, which has been successfully treated/prevented using iodised salt. The authors suggested that residual iodine deficiency in the population may have been due to a significant loss (48%) of iodine during transportation, improper storage at household level and highly variable iodine levels in the salt both at the point of production and consumption. In the present study, the KAP survey and shopkeeper survey included monitoring of salt storage at both these locations. It appeared that the salt was being stored appropriately in opaque packaging or jars to prevent loss of iodine through sunlight exposure. However, the testing of a small sample of local salt using rapid test kits revealed that none of salt samples analysed contained measurable quantities of iodine. A visit to a local salt producing factory highlighted poor quality control and standardisation of procedures for ensuring that the level of salt iodisation met the required standard. These findings indicate that improvements need to be made in the quality control of the salt iodisation process at in the salt factory. A more rigorous analysis of the iodine content of the salt sold locally and used in the home needs to be undertaken to confirm these observations.

There are some limitations to this study that need to be acknowledged. Firstly the sample size in the KAP survey was small. Due to time and budget constraints, only 50 of the original 1043 households were revisited. The KAP survey itself was thorough, including 68 semi-structured questions on a range of issues, which was designed to elicit similar responses from different questions, thereby triangulating the data. Secondly, the analysis of the salt for iodine was only undertaken in a small number of salt samples purchased from the bazaar. Ideally, salt should have been collected from the homes to determine the iodine content at point of use, and this activity is planned in the near future. Finally, the intervention and evaluation of impact were of short duration. It would be useful to assess the longer term impact of the intervention, and refresh with regular short campaigns if required.

## 5. Conclusions

This study has demonstrated that there is a clear need to improve the iodine status of the community living on the brick kilns in Peshawar. The levels of iodine deficiency in this community are unacceptable and will have a significant impact on the IQ and development of the children living here. A community designed education and awareness raising campaign regarding the benefits of iodised salt was very successful in terms of improving knowledge and increasing sales of iodised salt, however there may be serious challenges with the adequacy of the iodisation process at the point of production. Improving the iodisation procedures and quality control at local salt producing factories through support and training from technical experts is urgently recommended. Iodine losses during transport to remote areas need to be properly factored into the process and effective monitoring implemented. In addition, ensuring that there are no barriers to equitable access for all population groups, including the price which is an important concern in this low-income community.

## Supplementary Materials

Supplementary materials can be accessed at: http://www.mdpi.com/2072-6643/7/11/5490/s1.
