# Supplementary material for: Increasing Awareness and Use of Iodised Salt in a Marginalised Community Setting in North-West Pakistan
Source: Nutrients. 2015 Nov 23;7(11):9672–82. doi: 10.3390/nu7115490 (PMC4663618; doi:10.3390/nu7115490)
Supplement: Supplementary file 1 [file nutrients-07-05490-s001.pdf]

Supplementary Materials: Increasing Awareness and Use of Iodised Salt in a Marginalised Community Setting in North-West Pakistan

Nicola Lowe, Elizabeth Westaway, Akhtar Munir, Saba Tahir, Fiona Dykes, Monique Lhussier, Mick McKeown, Michael Zimmerman, Maria Andersson, Sara Stinca and Mukhtiar Zaman

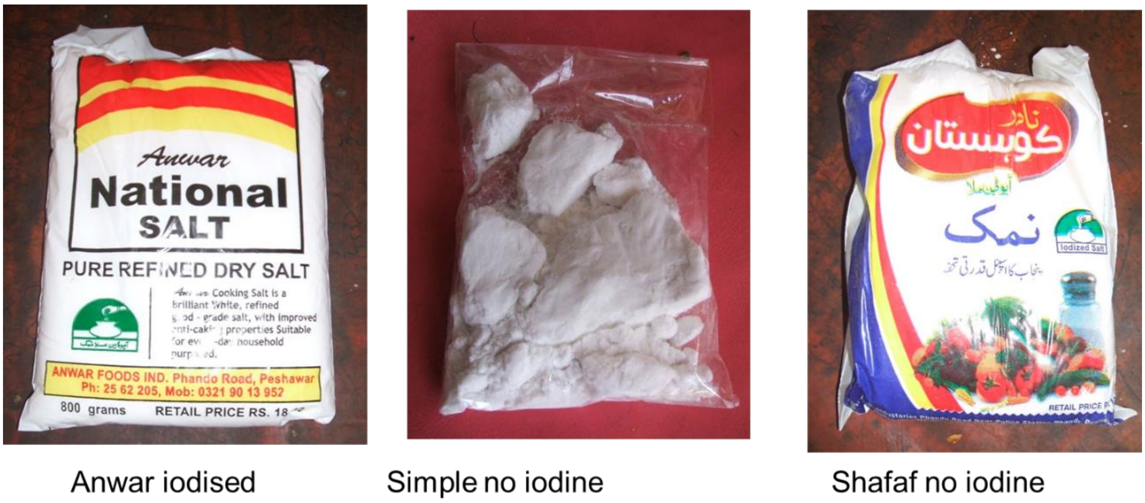

Figure S1. Salt available at the local Bazaar.

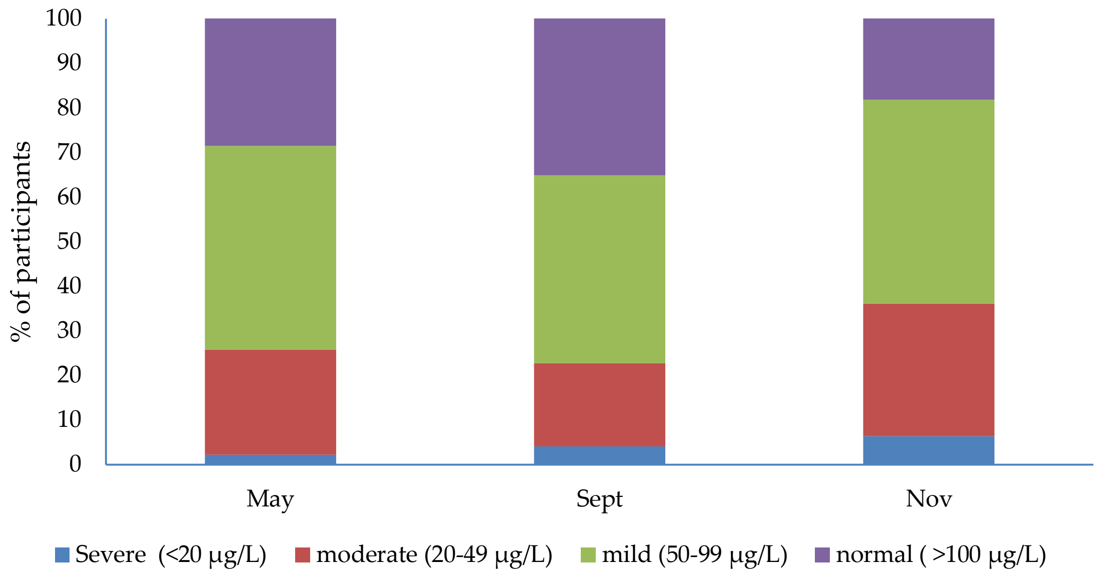

Figure S2. Distribution of the UICs between the population categories for iodine deficiency as defined by WHO.

Table S1. Characteristics of KAP survey respondents.

Characteristic	Number of Respondents (%)		
	All	Male	Female
Sex		25 (50)	25 (50)
Age group (years)			
25–35	2 (4)	0 (0)	2 (4)
36–45	12 (24)	2 (4)	10 (20)
46–55	18 (36)	7 (14)	11 (22)
>56	18 (36)	16 (32)	2 (4)
Can you read?			
Yes	0	0	0
No	50 (100)	25 (50)	25 (50)
Do you listen to radio?			
Yes	25 (50)	12 (24)	13 (26)
No	25 (50)	13 (26)	12 (24)
Do you watch Television?			
Yes	8 (16)	8 (16)	0 (0)
No	42 (84)	17 (34)	25 (50)
Did your son participate in the Urine Iodine analysis study?			
Yes	30 (60)		
No	20 (40)		

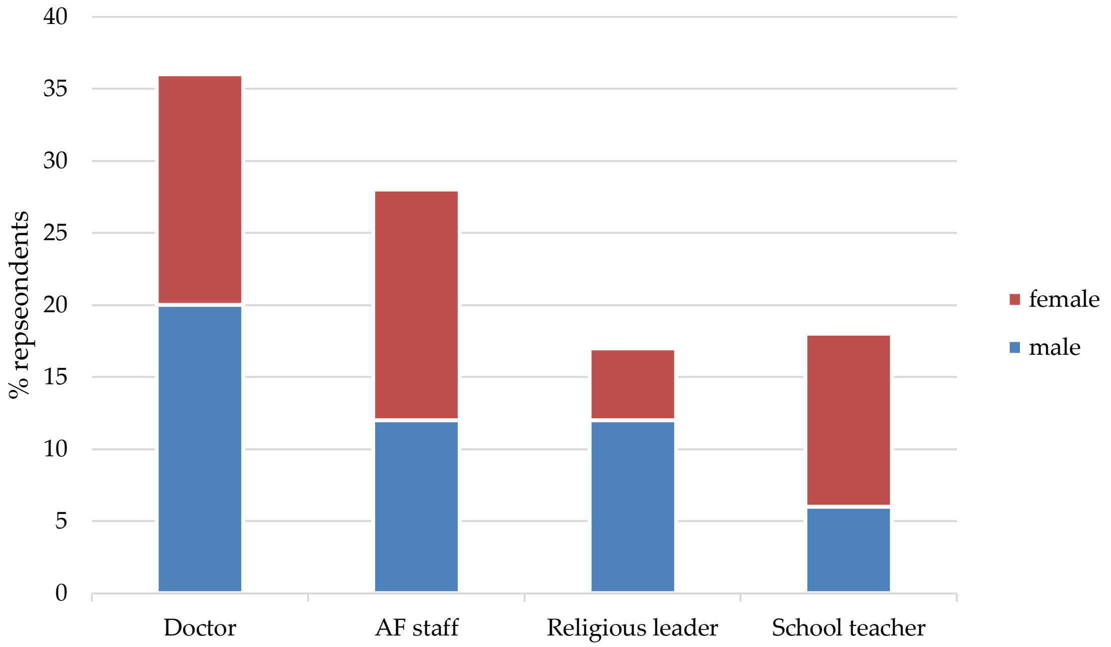

Figure S3. Who would you most believe in explaining to you the importance of iodised salt?

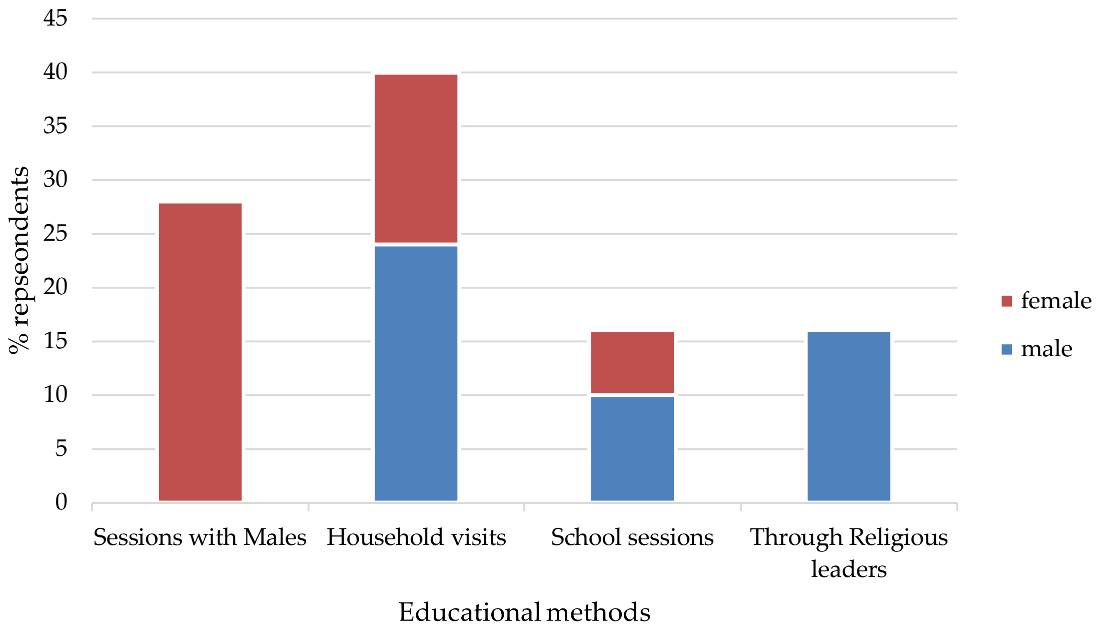

Figure S4. What are the best educational ways to make people believe iodised salt is important and essential for their health?
